# The effect of isolation methods of tomato pollen on the results of metabolic profiling

**DOI:** 10.1007/s11306-018-1471-4

**Published:** 2019-01-08

**Authors:** Marine J. Paupière, Yury M. Tikunov, Nurit Firon, Ric C. H. de Vos, Chris Maliepaard, Richard G. F. Visser, Arnaud G. Bovy

**Affiliations:** 1 Plant Breeding, Wageningen University & Research, PO Box 386, 6700 AJ Wageningen, The Netherlands; 2 Institute of Plant Sciences, The Volcani Center, ARO, Bet Dagan, Israel; 3 Bioscience, Wageningen University & Research, PO Box 386, 6700 AJ Wageningen, The Netherlands

**Keywords:** Pollen, Metabolome, Metabolomics, Metabolite, Anther, Tomato

## Abstract

**Introduction:**

Untargeted metabolomics is a powerful tool to detect hundreds of metabolites within a given tissue and to compare the metabolite composition of samples in a comprehensive manner. However, with regard to pollen research such comprehensive metabolomics approaches are yet not well developed. To enable isolation of pollen that is tightly enclosed within the anthers of the flower, such as immature pollen, the current pollen isolation protocols require the use of a watery solution. These protocols raise a number of concerns for their suitability in metabolomics analyses, in view of possible metabolic activities in the pollen and contamination with anther metabolites.

**Objectives:**

We assessed the effect of different sample preparation procedures currently used for pollen isolation for their suitability to perform metabolomics of tomato pollen.

**Methods:**

Pollen were isolated using different methods and the metabolic profiles were analysed by liquid chromatography–mass spectrometry (LC–MS).

**Results:**

Our results demonstrated that pollen isolation in a watery solution led to (i) rehydration of the pollen grains, inducing marked metabolic changes in flavonoids, phenylpropanoids and amino acids and thus resulting in a metabolite profile that did not reflect the one of mature dry pollen, (ii) hydrolysis of sucrose into glucose and fructose during subsequent metabolite extraction, unless the isolated and rehydrated pollen were lyophilized prior to extraction, and (iii) contamination with anther-specific metabolites, such as alkaloids, thus compromising the metabolic purity of the pollen fraction.

**Conclusion:**

We conclude that the current practices used to isolate pollen are suboptimal for metabolomics analyses and provide recommendations on how to improve the pollen isolation protocol, in order to obtain the most reliable metabolic profile from pollen tissue.

**Electronic supplementary material:**

The online version of this article (10.1007/s11306-018-1471-4) contains supplementary material, which is available to authorized users.

## Introduction

Over the last two decades new technologies have arisen which allow the examination of hundreds to thousands of molecules within a single analysis. Those breakthrough technologies called “Omics” have changed the way research is performed and led to a more comprehensive view of biochemical processes. Metabolomics platforms are used to measure the relative abundance of a large range of metabolites in any given tissue. More than 200,000 different metabolites are estimated to exist in the plant kingdom, a large proportion of which is specific to a certain species, genotype, tissue or condition. Hence, each biological sample has its own unique biochemical phenotype which can be determined using metabolomics. Association of genotypic, metabolic and phenotypic information led to an increased understanding of the genetic and metabolic basis for complex quality traits, such as aroma, colour or nutritional value, or biotic and abiotic stress resistance, and the development of genetic and metabolic markers to select for these traits (Tikunov et al. 2005; Riedelsheimer et al. 2012; Wahyuni et al. 2013; Viquez-Zamora et al. 2014; Xu et al. 2013). Metabolomics analyses generally include the following five major steps: (i) sample preparation, (ii) sample analysis, preferably using different analytical platforms, such as Gas Chromatography and Liquid Chromatography coupled to Mass Spectrometry (GC–MS and LC–MS, respectively), (iii) data processing, including peak picking, peak alignment and compound annotation, as far as is possible, (iv) statistical analysis and (v) data interpretation (Fiehn 2002).

In general, in order to compare the abundance of any metabolite in a set of biological samples it is essential to accurately determine the amount of sample and use the same sample weight per volume of extraction solvent. Therefore, usually between 10 mg dry weight up to 1 g of fresh material, well powdered in liquid nitrogen, is used for a single analysis. However, when working with small plant organs or tissues, such as pollen grains or trichomes, it can be highly challenging to obtain this amount of material and determine its exact weight. For instance, the diameter of one pollen grain from tomato (*Solanum lycopersicum*), is only about 20 µm and pollen of at least 10 mature flowers are needed to obtain 4 mg of (dry) tomato pollen.

In plants, pollen is a key organ for a successful sexual reproduction. Its role is to deliver its two sperm cells to the female gametophyte in order to fertilise an ovule. Developing pollen is very sensitive to environmental stresses, which can lead to a reduction of pollen quality and a concomitant decrease of yield in many crops. The importance of this issue is illustrated by the rapidly growing number of studies on pollen [e.g. tomato (Firon et al. 2006), sorghum (Prasad and Djanaguiraman 2011), and rice (Saragih et al. 2013)]. To understand the dynamic processes and key steps leading to the development of a mature and fertile pollen grain, several studies focussed on the analysis of transcripts, proteins and metabolites in pollen (Muschietti et al. 1994; Paupière et al. 2014; Rutley and Twell 2015). However, metabolomics of pollen grains has not been investigated to the same extent as other omics technologies (Supplementary Data Fig. 1). So far, several studies have applied targeted analytical approaches to study specific classes of compounds, such as carbohydrates and polyamines, during pollen development or in relation to environmental stresses, such as high temperatures (Pressman et al. 2002; Firon et al. 2006; Falasca et al. 2010) and only two studies have been performed to unravel the metabolic composition of developing pollen using untargeted metabolomics approaches (Paupière et al. 2017; Rotsch et al. 2017). The extension of such targeted analyses with comprehensive untargeted metabolomics approaches will contribute to a better understanding of the metabolic dynamics occurring in pollen during its development and in response to environmental stresses.

The key to obtain reliable metabolic information of pollen is the development and implementation of reliable methods and protocols for pollen sample preparation and metabolite extraction. As mentioned in a recent review by Nagele et al. (2017), available protocols for pollen isolation raise a number of concerns with regard to the metabolome, which need to be addressed in order to ensure their suitability for untargeted metabolomics analyses and interpretation of data. The most easy and direct manner to isolate pollen is by vibrating the flowers, leading to the release of mature dry pollen (Song et al. 2001, 2002). However, flower vibration does not allow the isolation of pollen at earlier stages of development, since these are still too firmly attached to the anther tissue. In order to study pollen at different developmental stages, pollen are commonly isolated by cutting anthers of different sizes (i.e. different developmental stages) into pieces with a razor blade and subsequently releasing their pollen by squeezing the anthers in an isotonic solution. Although this is the most widely-used method for pollen isolation (Aouali et al. 2001; Honys and Twell 2004; Firon et al. 2006; Castro and Clement 2007; Chaturvedi et al. 2013), it poses several concerns. Firstly, the isotonic solution consists of water, supplemented with mannitol or salts to prevent bursting of the pollen during their isolation (e.g. Honys and Twell 2004; Firon et al. 2006). However, during their maturation the pollen grains dry within the anthers. Thus, upon extraction from the anthers using a watery solution, the pollen may partially or fully rehydrate. This could lead to unwanted activation of enzymes present in the pollen which consequently may affect the metabolic content and, thus, this pollen isolated in water may no longer reflect its dry mature state. For instance, tomato pollen are known to contain an active acid invertase that may lead to conversion of sucrose into glucose and fructose during isolation of pollen in solution (Pressman et al. 2002). Secondly, to release pollen from the stamen, anthers are cut and/or squeezed. Although filters and washing steps are often used to prevent contamination from the surrounding tissue, these measures cannot fully prevent the release of metabolites from the anthers into the isolation solution.

The objective of this study was to assess the suitability of different sample preparation procedures currently used for pollen isolation, for metabolomics analyses of tomato pollen. We used three liquid chromatography-based analytical platforms, i.e. HPLC-LTQ Orbitrap MS of aqueous-methanol extracts, HPLC-Q Exactive Orbitrap MS of polar extracts and Dionex HPLC-ECD with electrochemical detection, in order to detect semi polar compounds, organic acids/amino acids, and sugars, respectively. Based on the metabolomics profiles obtained, we provide insight into the benefits, limitations and pitfalls of various approaches for pollen isolation, lyophilisation and metabolite extraction. We provide recommendations to limit metabolic alterations during the whole process of sample preparation and analysis. The present results are not only relevant for future metabolomics studies on pollen, but also provide more insight into the biochemistry of pollen during its isolation with the methods currently used to harvest pollen for other analytical approaches, including targeted metabolite analyses, proteomics and transcriptomics.

## Materials and methods

### Plant materials and growth conditions

Seeds of the tomato (*S. lycopersicum*) genotype M-82 were obtained from the Tomato Genetics Resource Centre. M-82 plants were grown in the greenhouse of Wageningen University & Research (The Netherlands) at 25 °C during the day and 19 °C during the night under approximatively 13 h of natural day light.

### Pollen isolation methods

Two pollen isolation methods were applied: (i) dry isolation by flower vibration, and (ii) wet isolation by anther squeezing in isolation solution (Supplementary Data Fig. 2).

For the flower vibration method (i), pollen were directly isolated by vibrating the flower still attached to the plant, using a milk frother to let the dry mature pollen fall into a 1.5-mL Eppendorf tube. This method was used in most of the experiments, in particular for the samples isolated by (a) vibration and then directly lyophilised (VL) and the technical replicates of these VL pollen (VLT), (b) vibration-derived pollen incubated in isolation solution before lyophilisation (VS), and (c) vibration-derived pollen incubated in isolation solution and then lyophilised (VSL). For the squeezing method (ii), the flowers were detached from the plants and kept on a petri dish on ice before pollen isolation. This method was used for pollen samples isolated by squeezing in solution and then lyophilised (AL). An overview of the different samples used is shown in Supplementary Data Fig. 2 and Supplementary Data Table 1. VL and VSL samples were used to assess the metabolic changes induced by the presence of ice cold isolation solution. VSL and VS samples were used to compare the impact of lyophilisation. AL and VSL samples were used to analyse metabolite contamination from the anthers. VLT samples were used to determine the technical reproducibility of the metabolite extraction and metabolomics profiling of exactly the same pollen material.

The isolation solution used to isolate pollen is described by Firon et al. (2006). The solution consisted of 1 mM KNO_3_, 3 mM Ca(NO_3_)_2_, 0.8 mM MgSO_4_ and 1.6 mM H_3_BO_3_ in distilled water. In both VSL and VS samples, 500 µL of ice cold solution was added to the 1.5-mL Eppendorf tube containing the mature pollen, followed by vortexing for 1 min and then keeping on ice for 1 h, corresponding to the approximate time necessary to isolate pollen in a standard experiment (data not shown). Eppendorf tubes were then centrifuged at minimum speed of 100×*g* for 2 min at 4 °C, followed by short spin at maximum speed of 17,000×*g* to spin down the pollen as a pellet. 400 µL of the supernatant was removed by pipetting and the remaining sample was frozen in liquid nitrogen and stored at − 80 °C.

For AL samples, pollen were isolated using an adapted version of the protocol described by Firon et al. (2006). In short, sepals and pistil were removed from the flower, after which the anther cone was cut into three pieces with a sharp razor blade and transferred into a 50-mL falcon tube on ice. Then, 10 mL of ice cold solution was added and the anthers were gently squeezed against the falcon tube wall with a 13-mL Sarstedt tube before to be vortexed for 10 s to allow pollen release. The liquid was filtered with two layers of miracloth Calbiochem® and transferred into a clean 50-mL falcon tube on ice. The sample was centrifuged at a minimum speed of 8×*g* for 15 min at 4 °C. During the centrifugation, the layers of miracloth were kept enclosed in the top of the tube, in order to recover the solution present in the miracloth. The supernatant was discarded and the pollen pellet was washed again with 500 µL of ice cold solution to limit anther contamination. The sample was shortly vortexed and the pollen suspension was transferred into a new 1.5-mL Eppendorf tube which was centrifuged at minimum speed of 100×*g* for 2 min at 4 °C followed by short spin. 400 µL of supernatant was removed by pipetting and samples frozen in liquid nitrogen and stored in − 80 °C.

Lyophilisation for 72 h was used to correct for differential water contents between samples.

### Homogenizing of pollen material

AL samples were used to test different pollen homogenization methods for metabolite extraction. Two different methods were used to grind pollen: (i) manual grinding with a pestle (pestle samples) and (ii) mechanical grinding with a tissue-lyser II Qiagen. Lyophilised mature pollen were used for each condition. For the pestle samples, pollen were ground with a polypropylene Eppendorf micro pestle in liquid nitrogen, the pollen powder was transferred in a 1.5-mL Eppendorf tube. 210 µL of 100% methanol and 90 µL of distilled water were added to the 1.5-mL Eppendorf tube to reach a final concentration of 70% methanol. For the tissue-lyser samples, methanol was added similarly as described above and three 2-mm stainless steel beads were added to the Eppendorf tubes. Pollen were subsequently ground with a tissue-lyser for 5 min, 10 min or 15 min, with a frequency of 25 Hz. From each homogenized extract a 10 µL aliquot was loaded into a Fuchs-Rosenthal haemocytometer (W. Schreck Hofheim/Ts) to count pollen grains that were still intact. Extracts were inspected under a light microscope and pollen grains were scored as still intact when they had a round shape with no visible bursts.

### Metabolite extraction for VL, VLT, VSL and AL samples

The preparation of both polar and semi-polar metabolite extracts from pollen material was carried out at room temperature using a protocol adapted from Wahyuni et al. (2013) and Carreno-Quintero et al. (2012). Before the homogenization step described above, a 10 µL aliquot of the aqueous-methanol extract was transferred into a new 1.5-mL Eppendorf tube containing 500 µL of isolation solution for pollen counting. The pollen counting was performed as described above and the pollen number served to normalise the metabolic data. The presence of 70% methanol did not disrupt the pollen cells, which allowed the pollen counting. Then, pollen homogenization was performed as described above with a tissue lyser grinding for 15 min at 25 Hz. The ground sample was sonicated for 10 min and centrifuged at maximum speed of 17,000×*g* for 10 min. Then 200 µL of the methanol supernatant was transferred into a new 1.5-mL Eppendorf tube containing 200 µL of 70% methanol to avoid metabolite saturation. The extract was then filtered over a 0.2 µm polytetrafluoroethylene (PFTE) filter. The filtered extract was divided in three parts for LTQ Orbitrap LC-MS, Q Exactive LC-MS and HPLC-ECD analysis, respectively.

For the LTQ Orbitrap LC-MS, 100 µL of filtered extract was transferred into a 2-mL crimp glass vial with an insert and directly used for analysis.

For both the Q Exactive LC-MS and the HPLC-ECD, 80 µL of filtered extract was transferred into a new 1.5-mL Eppendorf tube. Then, 80 µL of distilled water and 40 µL of chloroform were added and the sample was mixed for 5 min followed by centrifugation at maximum speed of 17,000×*g* for 10 min. 100 µL of the supernatant was transferred into a new 1.5-mL Eppendorf tube for HPLC-ECD, while 55 µL of supernatant was transferred into a new 1.5-mL Eppendorf tube. Both aqueous-methanol extracts were dried overnight in a speed vacuum.

For the HPLC-ECD, the dried extract was re-suspended in 100 µL of distilled water by pipetting and vortexing. Then 100 µL of a ion exchange resin was added (van Arkel et al. 2012). The sample was mixed on a roller band at 1000 rpm for 5 min at room temperature. The mixture was centrifuged at maximum speed of 17,000×*g* for 5 min and 100 µL of supernatant was transferred into a HPLC vial.

For the Q Exactive LC-MS, the dried methanolic extract was re-suspended in 55 µL of distilled water by pipetting and vortexing. Then 50 µL was transferred into a 2-mL crimp glass vial with a 100 µL insert.

### Metabolic profiling

The LTQ Orbitrap LC-MS system was composed of a C18 column (Phenomenex), an Acquity HPLC connected to a photodiode array (PDA) detector (both from Waters) and an LTQ/Orbitrap hybrid mass spectrometer (Thermo) as previously described by van der Hooft et al. (2012). The ion source was set in negative ionization ion mode. For the measurements, 10 µL of sample was injected into the system. The Xcalibur program was used for data collection.

The Q Exactive Orbitrap LC-MS analyses were carried out with a Dionex Ultimate 3000 Series RS pump coupled with a TCC-3000RS column compartment and a WPS-3000RS auto sampler (Thermo Fisher Scientific, Waltham, MA). A Discovery HS F5-3 (Supelco: 150*2.1 mm, 3 µm particles) column was used for chromatographic separation at 40 °C. Mobile phase A consisted of water and mobile phase B of acetonitrile, both acidified with 0.1% formic acid. The gradient started with 0% B for 5 min and was increased from 0% to 25% in 20 min. Then the column was washed by increasing mobile phase B to 80% in 5 min and held constant for 3 min. Finally, the mobile phase returned to 0% B and maintained for 8 min to equilibrate the column. A flow rate of 0.1 mL/min and an injection volume of 5 µL was used. The detection of compounds was performed using a Q Exactive Plus mass spectrometer (Thermo Scientific). A heated electrospray ionization source (HESI-II) in positive/negative ionization mode switching was used for ionization. The ionization voltage was optimized at 3.5 kV for positive mode and 2.5 kV for negative mode; capillary temperature was set at 250 °C; the auxiliary gas heater temperature was set to 220 °C; sheath gas, auxiliary gas and the sweep gas flow were optimized at 36, 10 and 1 arbitrary units, respectively. Full scan data in both positive and negative mode was acquired at a resolving power of 35,000 FWHM at *m*/*z* 200. A scan range of *m*/*z* 90-1350 was chosen. The automatic gain control was set at 3e6 and the injection time was set to 200 ms. External mass calibration was performed in positive and negative modes before each sample series. The Xcalibur software was used for data collection.

The HPLC-ECD (Dionex) was composed of a Carbopac™ PA-100 guard column, a Carbopac™ PA-100 (4 × 250) column and an ED40 Electrochemical detector (Dionex). The sugar elution was previously described by van Arkel et al. (2012).

### Metabolomics data processing

For the LTQ Orbitrap LC-MS, data were processed using MetAlign software available from http://www.metalign.nl; (Lommen 2009), to correct for baseline, peak picking, and mass alignment of chromatograms as previously described by Tikunov et al. (2005) and De Vos et al. (2007). The mass peaks were extracted and aligned by MetAlign sofware at a signal cut-off threshold setting of 12,500 ion counts per scan. Processed mass signals were kept for further analysis when they were present in at least all the biological replicates per treatment. MSClust software (Tikunov et al. 2012) was then used to group mass features originating from the same molecule and to extract quantitative ions of compounds represented by the highest intensity ion within the feature group. If a quantitative ion selected by MSClust showed saturation of the MS detector, this ion was replaced by its second or third isotopic ion. Putative annotation of metabolites detected was performed using an in-house accurate mass/retention time database generated by previous LCMS experiments on tomato tissues (Moco et al. 2006; van der Hooft et al. 2012) and the online database METLIN (http://metlin.scripps.edu/). The level of identification was performed according to The Metabolomics Standards Initiative requirements (Sumner et al. 2007): identified compounds got level I when Nuclear Magnetic Resonance was previously performed or an authentic standard has been used for unambiguous identification, level II when no authentic standard was used but annotation was made with both physicochemical property and spectral similarities, level III when the (class of the) compound has previously been reported for tomato, and finally level IV in case further annotation of the detected metabolite was impossible. In addition to the annotated compounds one ion was selected from each non-annotated mass ion cluster (or a putative mass spectrum) produced by MSClust and was considered as a putative compound. A cluster specific mass ion had to have the highest Cluster membership*Maximum abundance value.

For the Q Exactive LC–MS authentic standards of the organic acids malic acid, succinic acid and the amino acids proline, serine, asparagine, glutamine, threonine, glutamate, lysine, histidine, arginine, GABA, valine, isoleucine, leucine, and phenylalanine were used to ensure unambiguous identification. Chromatograms were extracted with Xcalibur software. A processing method was established using a ICIS peak integration based on the authentic standards considering a mass (*m*/*z*) window of 5 ppm, the retention time, the smoothing points, baseline window, area noise factor and peak noise factor. The parameters were optimized for each detected metabolite. The height of the peak was then retrieved from the peak integration file.

For the HPLC-ECD (Dionex), sugars were identified and quantified (in mg/mL) using a calibration curve of authentic fructose, glucose and sucrose standards.

### Statistical analysis

We used between five and six biological replicates for VL, VSL, VS and AL samples and six technical replicates for TL samples. Each biological replicate consisted of a pool of 10 flowers from a single plant, resulting in the isolation of 4–8 mg of mature pollen. All the metabolites were normalised by pollen number which varied between 156 and 56. To determine which metabolites were different between conditions, a one-way anova per single metabolite was carried out on log2 transformed values using the IBM SPSS statistic software package 20 (http://www.ibm.com). Performing a large number of statistical tests can lead to an increase of type I errors (false positives). For instance, with a classical significance threshold alpha of 0.05 per single test and the number of tests equal to the number of detected metabolites (57), 0.05*57 ≈ 3 metabolites could be expected to be false positives when no real differences exist. Hence, due to this high number of variables, we adjusted the significance threshold alpha to 0.005. Metabolites showing significance in the one-way anova test were followed up using a Tukey’s HSD post hoc test (alpha = 0.05).

## Results and discussion

In the procedure from pollen isolation to the final analysis of analytical data, the following steps were distinguished and examined:

- Pollen isolation and release
- Lyophilisation
- Pollen homogenisation
- Technical reproducibility

Of the 98 putative metabolites detected, we were able to annotate 56 metabolites including semi-polar secondary metabolites detected by the HPLC-LTQ Orbitrap MS (Supplementary Data Table 2), amino/organic acids detected by HPLC-Q Exactive Orbitrap MS (Supplementary Data Table 3) and sugars by Dionex HPLC (Supplementary data table 4). Although our analyses focussed primarily on annotated compounds, data for all detected putative compounds are shown in Supplementary Data Table 7.

It is worth mentioning that the generally applied Gas Chromatography–Mass Spectrometry platform to detect polar derivatized primary metabolites could not be used for the analysis of samples isolated in germination medium, since the presence of salts in the pollen isolation solution resulted in a significantly decreased TMS-derivatization efficiency (data not shown). Instead, we analyzed and compared amino/organic acids using HPLC-Q Exactive MS and sugars by Dionex HPLC. From the compounds detected by the three analytical platforms, only the early-eluting metabolites malate and arginine, both detected by the HPLC-Q Exactive MS platform, showed a decreased peak intensity in the presence of the salty pollen isolation solution, most likely due to ion suppression caused by co-eluting salts present in the injection peak (data not shown). Therefore, the interpretation of differential pollen isolation effects on these two compounds must be addressed with care.

### Pollen rehydration during isolation

**Fig. 1:**
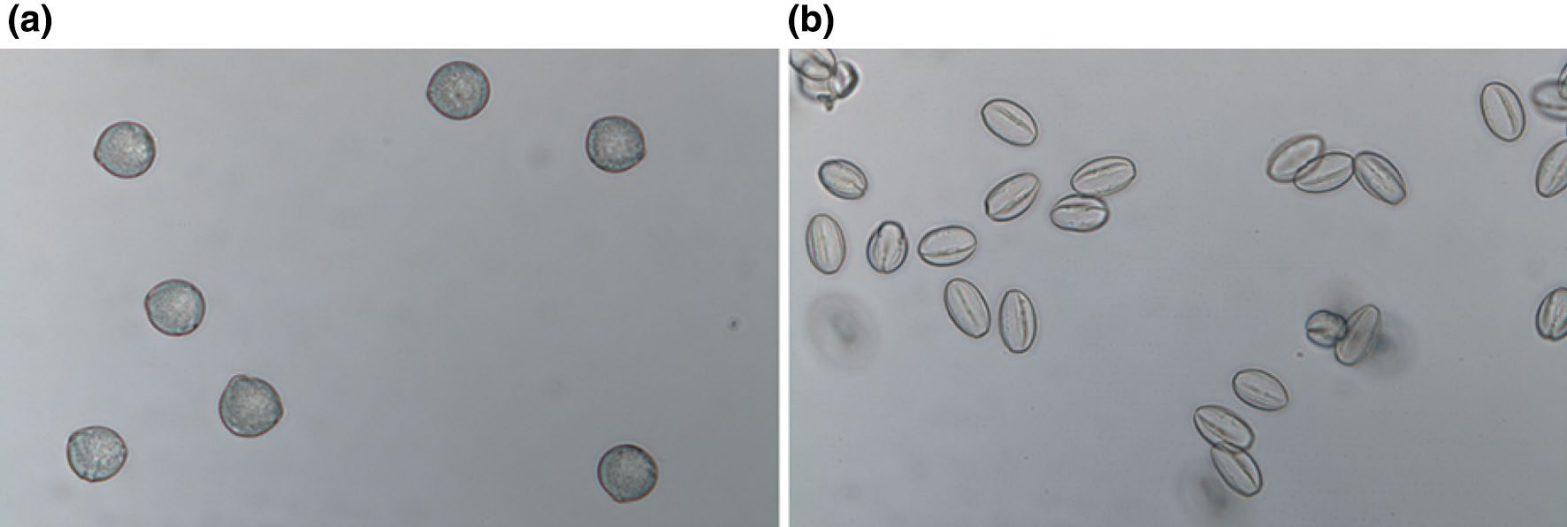
Pollen shape. Pictures of imbibed (**a**) and dry (**b**) mature pollen of M-82 observed under a light microscope. Dry pollen was observed in oil to avoid imbibition

**Fig. 2:**
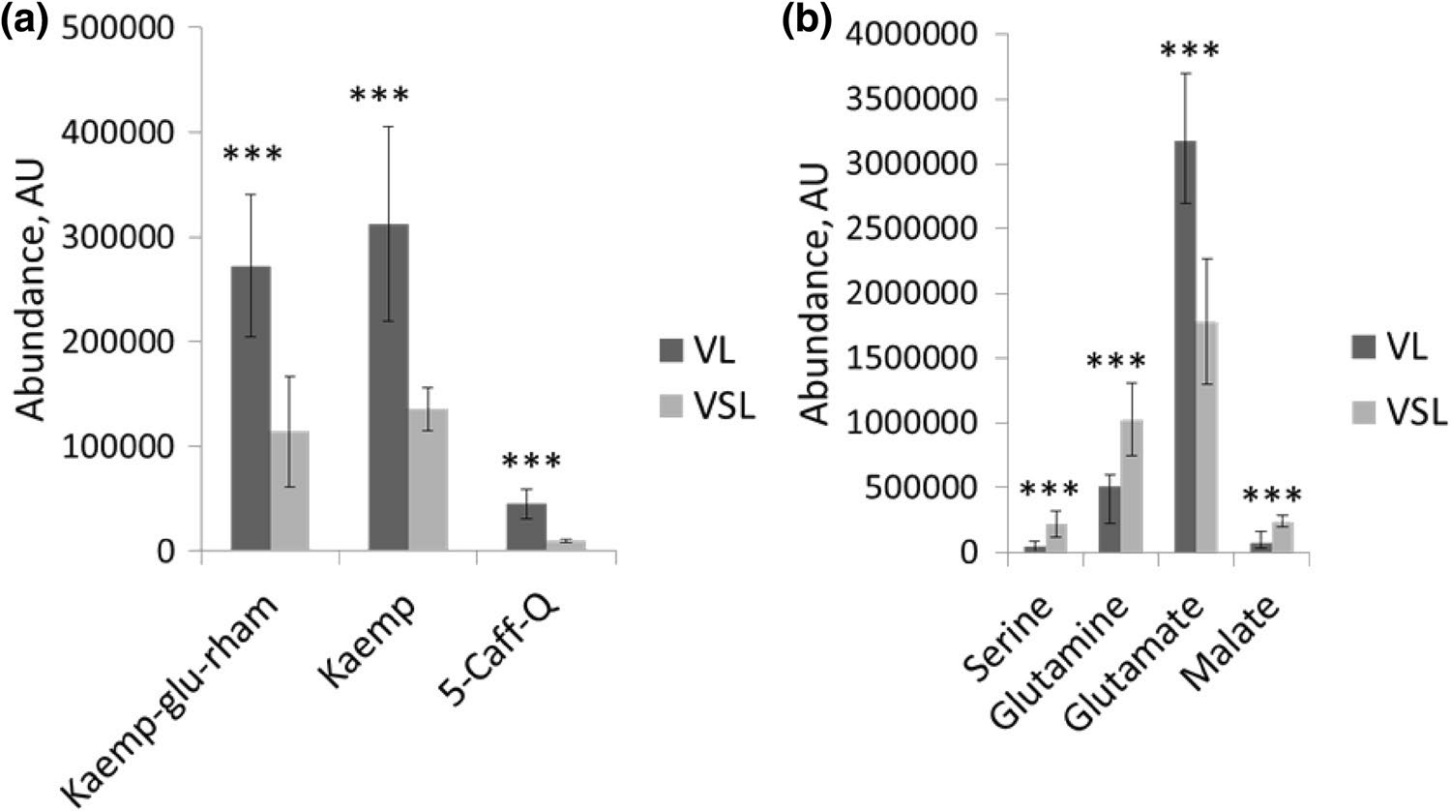
Metabolites affected by rehydration. **a** Compounds detected by the Orbitrap LC-MS method and **b** Compounds detected by Q Exactive LC-MS. *VL* vibrated pollen directly lyophilised, *VSL* vibration derived pollen firstly incubated in germination solution and then lyophilised. *Kaemp-glu-rham*: Kaempferol 3-alpha-d-glucoside-7-rhamnoside, *Kaemp* kaempferol, *5-Caff-Q* 5-Caffeoylquinic acid. Error bars represent the standard deviation among the observations (n = 5–6)

During pollen development, the pollen of tomato undergo a desiccation process in the final stage of their maturation, thereby conferring tolerance to abiotic stresses. When dry mature pollen were isolated in solution, the pollen underwent a rehydration process which was clearly visible under a light microscope (Fig. 1). Pollen increased their volume and obtained a round shape. To determine if these morphological transformations were accompanied with metabolic changes, VL and VSL samples were compared. Six metabolites, out of the total of 56 annotated ones, showed a significant difference of more than twofold between VSL and VL samples (Supplementary Data Tables 2 and 3). Two flavonoids (i.e. kaempferol aglycone and kaempferol-glucoside-rhamnoside) and the phenolic acid 5-caffeoylquinic acid were 2.3, 2.4 and 4.7-fold more abundant in VL samples than in VSL samples, respectively (Fig. 2a, b), whereas the amino acids serine, glutamine, and the organic acid malate were 4.7, 2.0 and 3.2-fold more abundant in VSL samples than in VL samples, respectively (Fig. 2b). This comparison indicates that isolation of mature pollen in solution, as compared to vibration derived pollen, leads to metabolic changes likely due to pollen rehydration.

Rehydration of pollen is known to have consequences on the metabolic dynamics. A study with *Capsicum annuum* pollen showed that only 30 min incubation in an osmotic solution at room temperature led to significant pollen rehydration and changes in sugar content (García et al. 2013). Furthermore, *Ricinus communis* seed, which, like pollen, is dry at maturity, showed marked metabolic changes upon rehydration at room temperature, with an accumulation of TCA metabolites (Ribeiro et al. 2015). In our experiments, we did not observe changes in sugar content in pollen collected and kept in cold pollen isolation solution, but the rehydrated pollen contained a larger amount of malate. Since we also observed that the detection of malate can be hindered by co-eluting salt components of the isolation solution, the increase in malate content in the rehydrated VSL samples, as compared to the dry VL samples, might even be an underestimation. In addition, dry pollen contained more glutamate, while imbibed pollen contained more glutamine. Since glutamate is the precursor of glutamine, we speculate that pollen rehydration led to a conversion of glutamate into glutamine by a yet unknown physiological mechanism. Although we performed the isolation of pollen on ice, in order to inhibit enzymatic activities as much as possible, these results suggest that this 1-h pollen incubation in solution, i.e. the approximate time needed to isolate (ripe or unripe) pollen from anthers, is sufficient to allow significant enzyme-mediated metabolic changes in the pollen. Support for metabolic activities even at low temperatures can also be drawn from the proteomics study on imbibing *Glycine max* seeds, in which an imbibition at 4 °C for 24 h showed marked changes in the protein profiles (Cheng et al. 2010). In addition to malate and glutamate/glutamine, several other compounds were altered by the 1-h incubation in isolation solution, such as flavonoids, a phenylpropanoid and the amino acid serine. The use of mature dry pollen without further contact with water (i.e. isolation using the flower vibration method) thus will optimally ensure the analysis of pollen in their actual biochemical state. However, pollen at earlier stages of development cannot be released by simply vibrating the flower, as immature tomato pollen are tightly enclosed in the anther locule. Hence, cutting and squeezing anthers in an osmotic solution is required if the research aim is to collect and compare pollen of different developmental stages. Based on the above, we recommend using the terminology ‘imbibed pollen’ when pollen isolation is performed in any water-containing solution, to discriminate with the data from naturally dry mature pollen. An interesting development is the use of vacuum devices to efficiently isolate dry pollen from anthers (King and Ferguson 1991; patent WO2012/5593A2). It remains to be established, however, whether such devices can also be used to isolate pollen fractions other than mature pollen in a dry manner, without the use of watery solutions and an anther squeezing step.

### Effect of pollen release from anthers

**Fig. 3:**
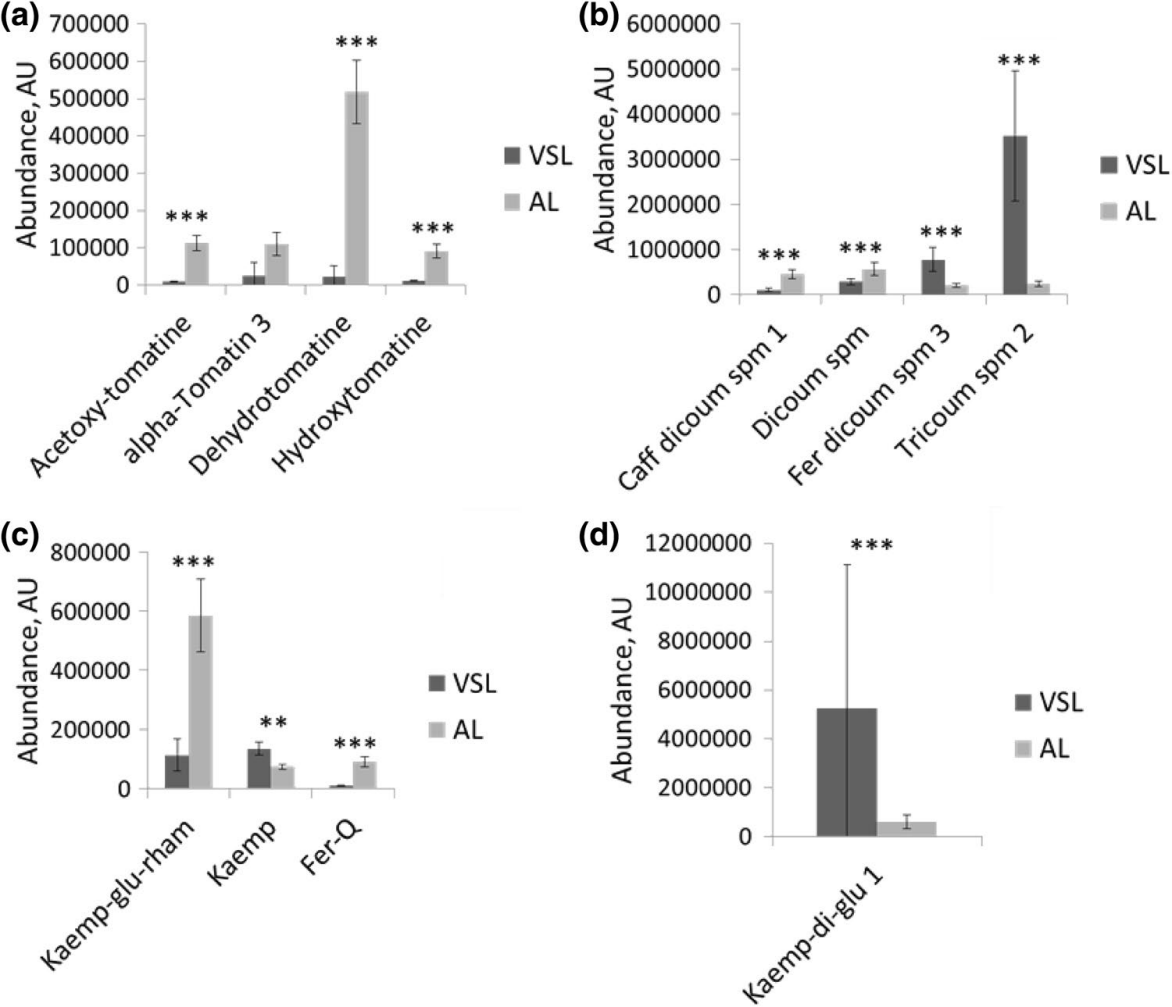
Metabolites affected by the release method. *VSL* vibration derived pollen incubated in germination solution and then lyophilised, *AL* pollen isolated by squeezing and then lyophilised. **a** Alkaloids; **b** polyamines; **c** flavonoids and phenolic acids; **d** flavonoid. *caff* caffeoyl, *dicoum* dicoumaroyl, *spm* spermidine, *fer* feruloyl, *tricoum* tricoumaroyl, *Kaemp* kaempferol aglycone, *glu* glucoside, *rham* rhamnoside, *Q* quinic acid. Error bars represent the standard deviation among the observations (n = 5–6)

When anthers are squeezed in a solution, contamination by anther tissue may take place (Supplementary Data Fig. 3), although these disrupted anther pieces are mainly removed by filtering through miracloth. Nevertheless, metabolites may be released from the anther tissues upon their squeezing in the isolation solution and thus contaminating the actual pollen metabolome. We therefore compared the metabolite profile of AL samples (isolated by anther squeezing) with the one of VSL samples (isolated by vibration with all other treatments being identical). All sugars, amino acids and organic acids, detected with both the Q Exactive LC-MS and the Dionex HPLC system, showed less than twofold differences between the two pollen release methods (Supplementary Data Tables 2 and 3). However, more than half of the annotated semi-polar secondary metabolites, detected by LTQ Orbitrap LC-MS, showed at least a twofold difference between the two release methods (Supplementary Data Table 2, Fig. 3). Most of the alkaloids as well as the phenylpropanoid feruloyl quinic acid present in the AL samples were not detectable in the VSL samples. In addition, several other compounds were significantly higher in AL compared to VSL samples, such as the flavonol glycoside kaempferol-glucoside-rhamnoside (5.1-fold), two forms of caffeoyl-dicoumaroyl spermidine (up to 4.2-fold) and dicoumaroyl spermidine (twofold) (Fig. 3b, c). Such an increase in the levels of several metabolites could reflect a contamination from anther fractions other than pollen cells. In contrast, the flavonoids kaempferol diglucoside 1 (8.4-fold), kaempferol aglycone (1.8-fold), three forms of the polyamines feruloyl-dicoumaroyl spermidine (up to 25-fold) and tricoumaroyl spermidine (15-fold) showed a higher accumulation in VSL samples compared to AL samples (Fig. 3b–d) and the flavonol quercetin-glucoside was detected in VSL samples only. Microscopic inspection of the AL samples revealed less than 1% anther contamination (results not shown) and it is therefore not expected that metabolite levels would strongly decrease due to anther contamination. In conclusion, these results indicate that the two different pollen isolation methods lead to samples with a markedly contrasting metabolite composition and suggest that other processes than anther contamination may also affect the metabolic composition of obtained pollen using these two isolation procedures. Recently, Fragkostefanakis et al. 2016 mentioned that the collection of pollen from anthers can be sorted in two categories: the released pollen, that is easily released by simple vortexing of the anthers in a solution, and the unreleased pollen, which represents the pollen grains that remain on the anther wall after vortexing and need mechanical disruption (squeezing) for their release. This distinction makes us speculate that the two releasing methods applied in our study, VSL and AL, may lead to isolation of different pollen types, analogous to the released and unreleased pollen fractions mentioned by Fragkostefanakis et al. (2016). We cannot exclude that our AL samples include “unreleased” pollen, in addition to the released pollen. The latter may metabolically differ from the fraction of released pollen, which is likely the major fraction in the VSL samples. The difference of pollen fraction might also explain the strong difference in the presence of alkaloids. However, we previously assessed the metabolic composition of released pollen isolated with cut anthers in solution, but without the squeezing step, and observed similar alkaloid accumulation. This implies that alkaloids are more likely to come from the anther fraction than from a different pollen population. Besides, several studies have also shown that metabolites can be found at the surface of the pollen, such as flavonoids and polyamines (vanTunen et al. 1991; Grienenberger et al. 2009). The filtration and washing step used to isolate AL samples could lead to a decrease of these specific metabolites. In conclusion, the pollen isolation method based on squeezing of anthers in germination medium should be considered with care, since it could lead to anther contamination, isolation of different pollen fractions or washing away of surface compounds, in addition to the above-mentioned metabolic effect of imbibition. Recently, the metabolic profile of developing pollen has been studied in tobacco (*Nicotiana tabacum*) (Rotsch et al. 2017) and tomato (Paupière et al. 2017) using GC-MS and LC-MS metabolomics platforms, respectively. Since anther squeezing was needed in these studies to release tightly enclosed microspores, the obtained results should be considered in view of the above mentioned methodological artefacts. In this respect it is noteworthy to mention that a new protocol for pollen isolation, using a Percoll gradient, showed promising improvements with respect to the purity of the sample (Nikoleta Dupl’áková et al. 2016). However, this method is time consuming (4–6 h) and could therefore easily lead to metabolic changes, considering that young microspores are metabolically active.

### The effect of lyophilisation on the metabolic profile of tomato pollen

The final step of the pollen isolation procedure in solution results in a pellet of imbibed pollen cells submerged in solution. There are two reasons to remove this solution and dry the pollen sample by lyophilisation: (1) pollen have different levels of water status during their development. Lyophilising samples is thus required to avoid possible metabolite differences related to differential water content of developmental stages; (2) pollen contains a very high invertase activity that may lead to conversion of sugars in an aqueous environment (Pressman et al. 2002). To test whether lyophilisation might help in preventing enzyme-related metabolic conversions during isolation of pollen in solution, dry pollen were isolated by flower vibration and incubated for 1-h in solution, to mimic the isolation in solution with squeezing, and subsequently frozen in liquid nitrogen. Half of the sample was dried by lyophilisation for 72 h before metabolite extraction (VSL samples), while the other half was extracted “wet” (VS). During tissue homogenisation and metabolite extraction, the ratio solvent/water used for lyophilised (VSL) and non-lyophilised samples (VS) was adjusted to correct for their differential water contents.

**Fig. 4:**
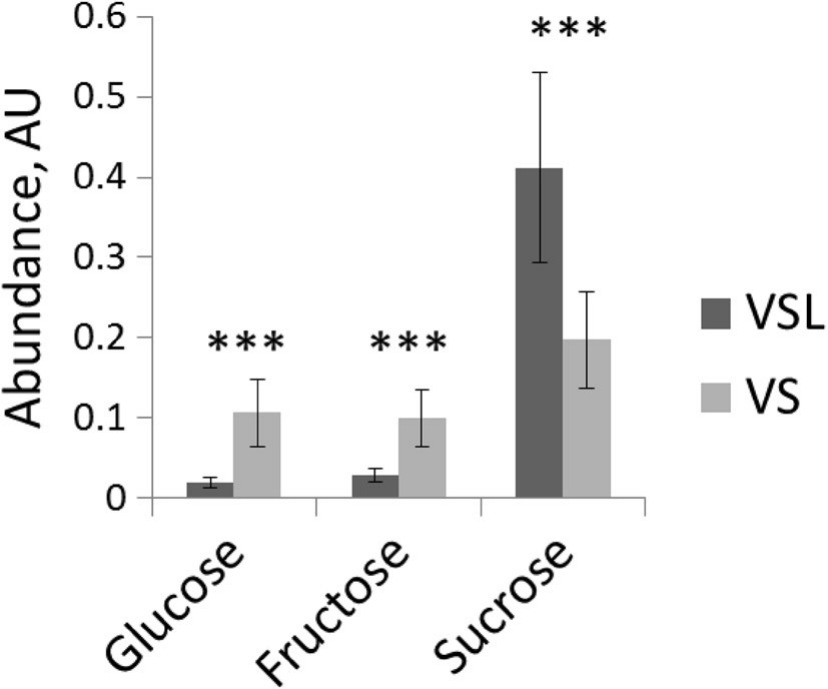
Metabolites affected by lyophilisation. *VSL* vibration derived pollen incubated in germination solution and lyophilised, *VS* vibrating pollen incubated in germination solution and non-lyophilised. Error bars represent the standard deviation among the observations (n = 5–6)

Among the annotated metabolites detected by the three metabolomics platforms, only the three sugars sucrose, fructose and glucose showed a statistically significant and more than twofold difference between the two samples (Supplementary Data Table 4). The hexoses glucose and fructose were present at 5.7 and 3.5-fold higher levels, respectively, whereas sucrose levels were twofold lower in VS samples compared to VSL samples (Fig. 4). We obtained similar results when pollen were isolated with the standard isolation protocol using the anther squeezing method (data not shown). These results revealed that lyophilisation of rehydrated mature or wet unripe pollen is an important step to avoid conversion of sucrose into hexose sugars by the action of acid invertases. These results also suggest that this conversion takes place after the pollen isolation step, i.e. during their homogenisation and/or extraction. In VS samples the mature pollen were wet and surrounded by solution, although frozen in liquid nitrogen, when pure methanol (at room temperature) was added that might thaw the tissue prior to tissue homogenization, while in VSL samples the pollen were dry before addition of methanol. We speculate that it was at this thawing step in methanol or during homogenisation when the acid invertase can be temporally activated at least in the case of the non-lyophilized pollen. Hence, we recommend to always lyophilise isolated pollen samples before metabolite extraction. As Obermeyer et al. (2013) have performed extraction of pollen metabolites of Lily (*Lilium longiflorum*) with methanol precooled at − 20 °C, adding a cold extraction solution can be an alternative to lyophilisation in order to prevent acid invertase activity. However, extraction at low temperatures needs further investigation, since it might also affect the extraction efficiency of less-soluble metabolites.

### The effect of pollen homogenization

**Fig. 5:**
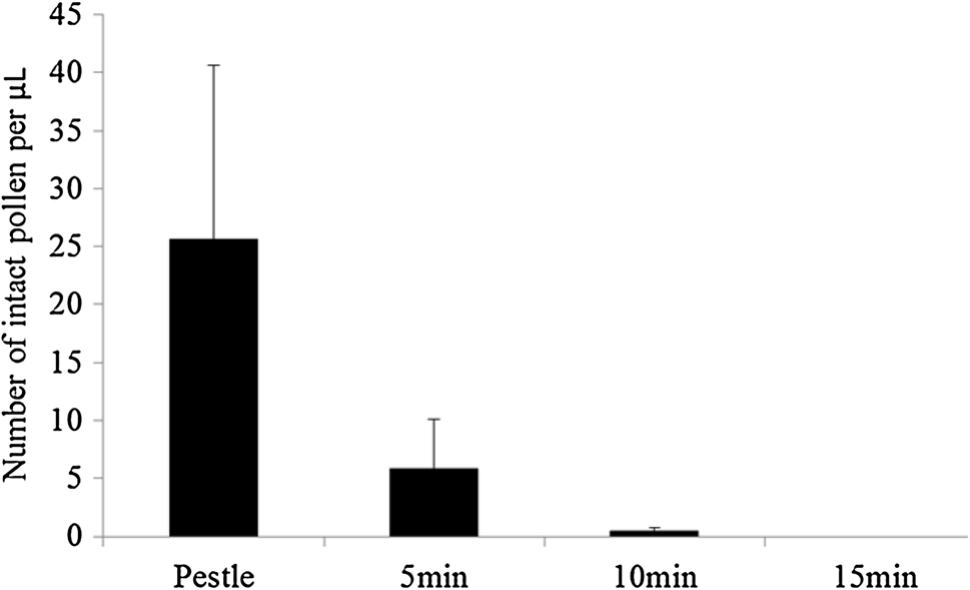
Number of intact pollen grains remaining after different grinding methods. Pestle: manual grinding with an Eppendorf micropestle; 5, 10, 15 min: grinding for indicated time with three stainless steel beads in an automatic tissue-lyser. Number of pollen grains remaining after treatments were counted under a light microscope. Error bar represents the standard deviation among the observations

Before performing a metabolomics analysis, it is recommended to grind the tissue in order to facilitate and optimize the release of metabolites into the extraction solvent. To determine the optimal method to obtain homogenized pollen material, we compared two different homogenization techniques for mature pollen: (ii) manual grinding using an Eppendorf micro pestle and (ii) mechanical grinding using a tissue-lyser. The latter was tested at increasing duration of grinding (Fig. 5). Subsequent counting of the number of intact pollen under the microscope (Fig. 5) revealed that the grinding with a micro pestle gave always more intact pollen per µL than with the tissue-lyser: intact pollen were clearly visible in the pestle samples, while grinding with the tissue-lyser for only 5 min already broke most pollen and after 15 min no intact pollen was observed at all. We previously observed that the presence of a high number of intact cells leads to a lower abundance of metabolites in the methanolic extracts (data not shown), suggesting that 70% methanol is not sufficient to open the intact pollen cells, due to the thick wall that surrounds the pollen. Hence, we recommend the use of a tissue lyser to grind the pollen for at least 15 min.

### Technical reproducibility

Dry pollen of different tomato plants were pooled and divided into six aliquots in order to assess the technical reproducibility of metabolite extraction of pollen tissue. In total 50 metabolites could be annotated in these pollen. The median of the coefficient of variation of these metabolites was 14% (Supplemental Data Table 5).

### Non-targeted metabolic analysis

Although our analyses focussed on annotated metabolites only, it can be expected that the metabolic effects of different sample preparation procedures is not restricted to these metabolites only. In addition to the annotated compounds we extracted a representative mass ion for each putative mass spectrum derived from the LC-MS profiles of semi-polar secondary metabolites. A principal components analysis (PCA) of these untargeted data showed that the method of pollen extraction from the anther had the largest effect on the overall secondary metabolite composition and was captured in the first principal component (53% of the total variation) (Supplemental Data Fig. 4). 50 of 79 secondary metabolites − 63% of the total representative metabolic composition—had a moderate-to-high correlation (R > 0.4) with this principal component (Supplemental Data Table 6). In addition, 16% of the variation could be explained by the use of the germination solution after the pollen were extracted from the anthers. 34% of the compounds correlated with the principal component which captured the effect of germination solution treatment (27 of 79 with R > 0.4). This analysis clearly shows that the metabolic effects of the different treatments are not restricted to the 56 annotated compounds, but may also affect a considerable proportion of the total pollen metabolome. Annotation of such metabolites may shed more light on the unintended effects of different sample preparation methods. This however, was not part of this study.

## Conclusions

We have shown that different steps in the pollen isolation and extract preparation protocols usually applied in transcriptomics and proteomics research significantly influence the tomato pollen metabolome. To summarize we recommend (i) the use of a tissue lyser for grinding pollen for at least 15 min to ensure the optimal breaking of pollen cells, (ii) to use only pollen isolated by vibration to study the metabolic composition of mature pollen, (iii) if mature pollen are to be compared with earlier stages of development, an isolation solution is needed and the pollen should thus be qualified as imbibed, (iv) more efforts should be put on finding an isolation solution that prevents pollen rehydration. However, the suitability of any new isolation solution for metabolite extraction and metabolomics analysis needs to be assessed, (v) the matrix effect must be verified when samples contain salts as used in standard pollen isolation solution. Alternatively, a different isolation solution should be used to prevent matrix effects (i.e. mannitol supplemented water), (vi) the experimentalist needs to be aware that anther contaminations can occur during the squeezing step to release its pollen. Hence, the optimum method to isolate pollen is to use the VL method which keeps the pollen as much as possible in its biological state at harvest. More investigations are required to improve the purity of the pollen fraction in any isolation method, either by increasing the washing and filtration step, or by determining the metabolic specificity of each fraction. Although we made progress to achieve a reliable metabolic profile of pollen cells, several aspects still remain unanswered and deserve further investigations: how might pollen at young developmental stages react to the incubation in ice cold isolation solutions? How can we assess the purity of pollen samples? Do different fractions of pollen derived from the same anther differ metabolically? And why did the conversion of sugars not occur during pollen isolation even though that lasted for 1-h, but apparently did occur rapidly during extraction?

## Electronic supplementary material

Below is the link to the electronic supplementary material.

Supplementary material 1 (DOCX 13 KB)

Supplementary material 2 (DOCX 150 KB)

Supplementary material 3 (XLSX 233 KB)
