# Supplementary material for: The effect of isolation methods of tomato pollen on the results of metabolic profiling
Source: Metabolomics. 2019 Jan 8;15(1):11. doi: 10.1007/s11306-018-1471-4 (PMC6326007; doi:10.1007/s11306-018-1471-4)
Supplement: Supplementary file 1 — Supplementary material 1 (DOCX 13 KB) [file 11306_2018_1471_MOESM1_ESM.docx]

**Supplementary data table 1** Overview of samples used in this study

**Supplementary data table 2** Annotated metabolites detected by the Orbitrap LC-MS method. The table represent the annotation information per metabolite including compound class, retention time (ret.), negative mass, ∆ retention time, ∆ ppm of the mass, annotation level (Ann. Level. See materials and methods section), CID, compound ID from PubChem, reference (ref.) with 1, Moco et al. 2006;2,Iijima et al. 2008;3,Roldan et al. 2014;4,Ferreres et al. 2010;5, https://metlin.scripps.edu/index.php ;6,Yang et al. 2012; 7,Elejalde-Palmett et al. 2015 and the average per sample condition, in which AL means pollen isolated by squeezing and lyophilised; VL, vibration derived pollen lyophilised; VS, vibrating pollen incubated in germination solution and non-lyophilised; VSL, vibrating pollen incubated in germination solution and lyophilised, and the fold change between the different conditions (condition 1/condition 2), if the value is positive, condition 1 has a higher value than condition 2; if the value is negative, condition 1 has a lower value than condition 2. The ‘ indicate the significance level in the Tukey post-hoc test that followed the univariate ANOVA analysis with p < 0.005, '''; 0.005 <p< 0.01, ''; and 0.01 < p<0.05, '. d.m., data missing. Fold changes larger than 2-fold are bold and grey highlight

**Supplementary data table 3** Annotated metabolites detected by HPLC-Q Exactive Orbitrap MS. The table represent the annotation information per metabolite including compound class, retention time (ret.), CID, compound ID from PubChem, and the average per sample condition, in which AL means pollen isolated by squeezing and lyophilised;; VL, vibration derived pollen lyophilised; VS, vibrating pollen incubated in germination solution and non-lyophilised; VSL, vibrating pollen incubated in germination solution and lyophilised, and the fold change between the different conditions (condition 1/condition 2), if the value is positive, condition 1 has a higher value than condition 2; if the value is negative, condition 1 has a lower value than condition 2. The ‘ indicate the statistic threshold on the Tukey post-hoc test that followed the univariate ANOVA analysis with p< 0.005, ''' ; 0.005 <p< 0.01, ''; and 0.01 < p<0.05, '. d.m., data missing. Fold changes larger than 2-fold are bold and grey highlight

**Supplementary data table 4** Annotated metabolites detected by Dionex HPLC. The table represent the average per sample condition, in which AL means pollen isolated by squeezing and lyophilised; VL, vibration derived pollen lyophilised; VS, vibrating pollen incubated in germination solution and non-lyophilised; VSL, vibrating pollen incubated in germination solution and lyophilised, and the fold change between the different conditions (condition 1/condition 2), if the value is positive, condition 1 has a higher value than condition 2; if the value is negative, condition 1 has a lower value than condition 2. The ‘ indicate the statistic threshold on the Tukey post-hoc test that followed the univariate ANOVA analysis with p < 0.005, ''' ; 0.005 <p< 0.01, ''; and 0.01 < p<0.05, '. d.m., data missing. Fold changes larger than 2-fold are bold and grey highlight. CID, compound ID from PubChem.

**Supplementary data table 5** Technical variation of the metabolomics analyses. For all annotated metabolites, technical replicates (n=6) were analysed and the variation was expressed as coefficient of variation (CV; column J).

**Supplemental data table 6** Correlations of the semi-polar metabolites to the first two principal components of PCA of Supplemental data Fig. 4. High positive correlation means that a metabolite increased in concentration in response to the pollen treatment captured by a principal component, or decreased in concentation if correlation is negative. Names of annotated metabolites correspond to the names in Supplemental data Table 2. Unknown metabolites are labelled as e.g. “Unk_1.44_158.9785” where the first and the second number represent the retention time and the mass [M-H]^-^, respectively.

**Supplemental data table 7** Total list of mass ions and their abundances detected in all the pollen extracts analysed in the study. All mass ions are classified into putative mass spectra (mass ion clusters) (column A) and their membership value in the corresponding clusters are provided (column B), followed by Retention time, min (C), negative ion mass, Da (D), compound name or a selected ion code (E), annotated compound biochemical class (F), for the annotated compounds: the deviation of the experimental retention time from the library retention time (G), mass deviation (H), annotation level (I), CID identifier (J) and a literature reference (K).
