# Supplementary material for: The effect of isolation methods of tomato pollen on the results of metabolic profiling
Source: Metabolomics. 2019 Jan 8;15(1):11. doi: 10.1007/s11306-018-1471-4 (PMC6326007; doi:10.1007/s11306-018-1471-4)
Supplement: Supplementary file 2 — Supplementary material 2 (DOCX 150 KB) [file 11306_2018_1471_MOESM2_ESM.docx]

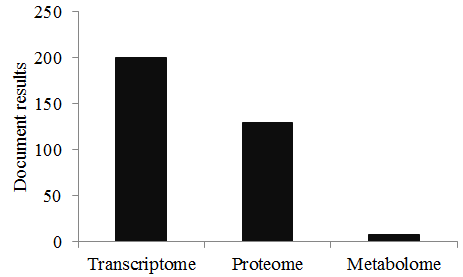


**Supplementary data Fig. 1** Numbers of documented results from a search of the Scopus data base ([www.scopus.com](http://www.scopus.com)) with the keywords “pollen transcriptome”, “pollen proteome” and “pollen metabolome”


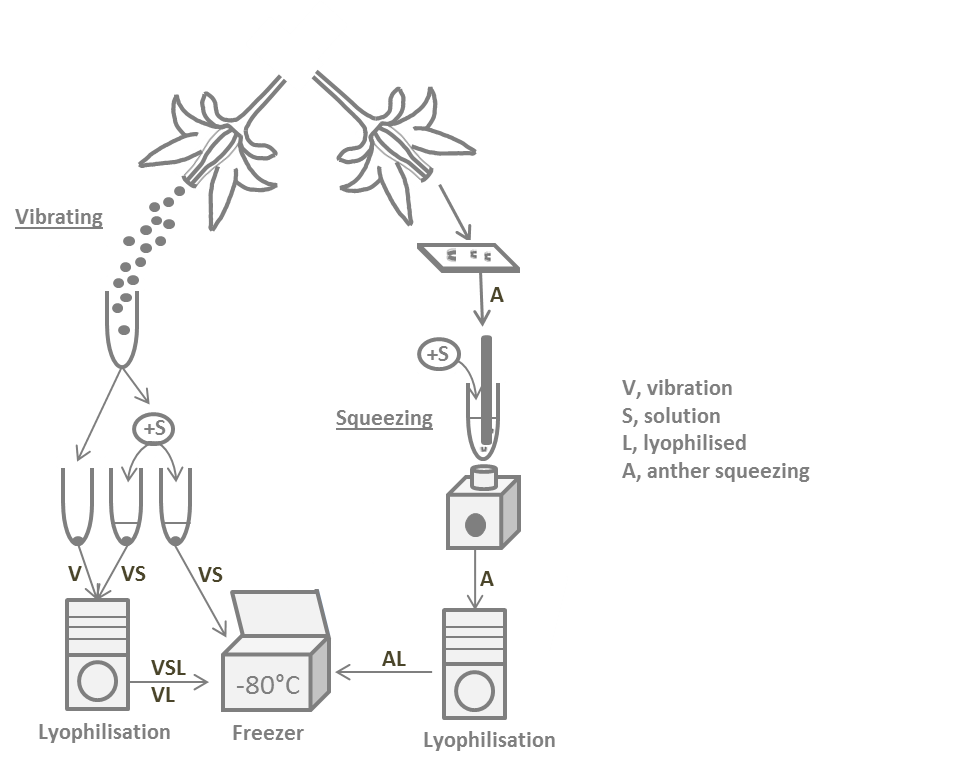


**Supplementary data Fig. 2** Schemetic of pollen isolation methods tested. VSL: sample isolated by vibration incubated for one hour in germination solution (+S) and lyophilised; VL: sample isolated by vibration and lyophilised; VS: sample isolated by vibration incubated for one hour in germination solution and non lyophilised; AL: sample isolated by anther (A) squeezing in germination solution and lyophilised


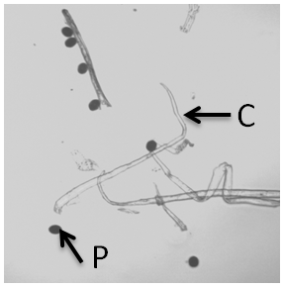


**Supplementary data Fig. 3** Contamination from anther tissue during pollen isolation. Pollen grains were stained with Alexander dye for a better visualisation under light microscope. P, pollen grain and C, anther contamination


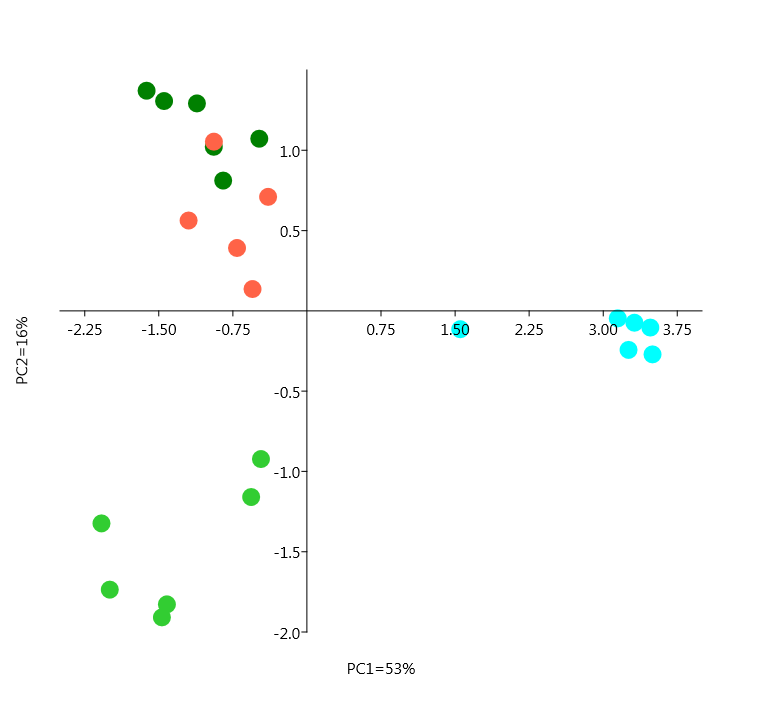


germination solution

vibration

squeezing

No germination solution

**Supplementary Data Fig. 4.** Principal Components Analysis (PCA) of tomato pollen based on untargeted metabolic profiles of 79 semipolar compounds determined using LC-MS. Light blue – squeezing/inc. in germination solution/lyophilized (AL), light green – vibration/no germination solution/lyophilized (VL), dark green – vibration/inc. in germination solution/no lyophilization (VS), red – vibration/inc. in germination solution/lyophilized (VSL). PCA showed that the first principle component (53% of total variation) captures the metabolic variation caused by the two different pollen isolation methods – squeezing from anthers and vibration of anthers and the second principal component (16%) seemed to separate the pollen extracts based on the use of germination solution. Correlations of the metabolites to the principal components can be viewed in Supplementary Data Table 7.
